# Trapalyzer: a computer program for quantitative analyses in fluorescent live-imaging studies of neutrophil extracellular trap formation

**DOI:** 10.3389/fimmu.2023.1021638

**Published:** 2023-06-08

**Authors:** Michał Aleksander Ciach, Grzegorz Bokota, Aneta Manda-Handzlik, Weronika Kuźmicka, Urszula Demkow, Anna Gambin

**Affiliations:** ^1^Faculty of Mathematics, Informatics and Mechanics, University of Warsaw, Warsaw, Poland; ^2^Centre of New Technologies, University of Warsaw, Warsaw, Poland; ^3^Department of Laboratory Diagnostics and Clinical Immunology of Developmental Age, Medical University of Warsaw, Warsaw, Poland

**Keywords:** neutrophil, neutrophil extracellular traps, fluorescent microscopy, digital image processing, image annotation, SYTOX™ green, chromatin, quantification

## Abstract

Neutrophil extracellular traps (NETs), pathogen-ensnaring structures formed by neutrophils by expelling their DNA into the environment, are believed to play an important role in immunity and autoimmune diseases. In recent years, a growing attention has been put into developing software tools to quantify NETs in fluorescent microscopy images. However, current solutions require large, manually-prepared training data sets, are difficult to use for users without background in computer science, or have limited capabilities. To overcome these problems, we developed Trapalyzer, a computer program for automatic quantification of NETs. Trapalyzer analyzes fluorescent microscopy images of samples double-stained with a cell-permeable and a cell-impermeable dye, such as the popular combination of Hoechst 33342 and SYTOX™ Green. The program is designed with emphasis on software ergonomy and accompanied with step-by-step tutorials to make its use easy and intuitive. The installation and configuration of the software takes less than half an hour for an untrained user. In addition to NETs, Trapalyzer detects, classifies and counts neutrophils at different stages of NET formation, allowing for gaining a greater insight into this process. It is the first tool that makes this possible without large training data sets. At the same time, it attains a precision of classification on par with state-of-the-art machine learning algorithms. As an example application, we show how to use Trapalyzer to study NET release in a neutrophil-bacteria co-culture. Here, after configuration, Trapalyzer processed 121 images and detected and classified 16 000 ROIs in approximately three minutes on a personal computer. The software and usage tutorials are available at https://github.com/Czaki/Trapalyzer.

## 1 Introduction

Neutrophils are the most abundant group of white blood cells in humans. They are often described as the organism’s “frontline soldiers”, responsible for fighting pathogens during the initial stages of infection (1, 2). One of their fighting strategies is the formation of Neutrophil Extracellular Traps (NETs), web-like structures formed from the cells’ DNA, which ensnare and putatively kill microbes (3, 4). NETs help to fight infections, but may also harm the host by damaging surrounding tissues and promoting inflammation (5). Research shows that excessive or insufficient formation of NETs plays a role in a number of diseases, including periodontitis, thrombosis, and arthritis (6, 7). A better understanding of the dynamics of NET formation may lead to improved diagnostics and treatment of those diseases. This requires both qualitative studies of the biology of this process as well as quantitative studies of its rates in different conditions.

### 1.1 The biology of NET formation

**Figure 1:**
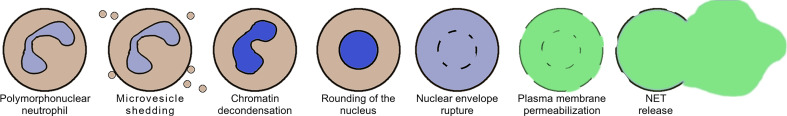
A schematic representation of the selected stages of Neutrophil Extracellular Trap (NET) formation, based on (8).

NET formation induced by either ionomycin or the presence of *Candida albicans* has recently been studied on a cellular level by the means of high-resolution time-lapse microscopy (8). The authors have observed that this process progresses through a sequence of stages, shown schematically in **Figure 1**. The onset of NET formation is marked by the disassembly of the actin cytoskeleton and the formation of plasma membrane microvesicles containing cytosolic components. Next, the neutrophils’ chromatin gradually decondenses, with its fluorescent staining becoming spatially homogeneous. During and after chromatin decondensation, the nucleus loses its characteristic lobulation and becomes partially or fully rounded. After some time, a rapid disruption of the nuclear envelope causes a release of the DNA into the cytoplasm. Simultaneously, the plasma membrane gradually increases its permeability, causing membrane-impermeable markers to enter the cell. Finally, the plasma membrane ruptures, releasing the genetic material to the environment.

### 1.2 Methods and technical challenges of computer-assisted NET quantification

In recent years, there has been a growing interest in developing computational methods of NET quantification to make it more replicable and objective, while at the same time less laborsome and time-consuming (9). A number of computer programs for NET quantification has been released, either based on machine learning algorithms, including convolutional neural networks and support vector machines (10–12), or digital image processing techniques, including image thresholding and classification of regions of interest (ROIs) based on features such as area or circularity (13–15). Modern machine learning-based methods are capable of quantifying not only NETs, but also cells at certain stages of NET formation, giving a greater insight into the dynamics of this process (12).

However, the complex nature of NET formation poses a substantial difficulty in developing software tools to analyze it. Furthermore, there are numerous experimental methods of NET quantification (7, 16, 17), and each experimental method not only requires a different computational approach, but also determines which stages of NET formation can be quantified. For example, microvesicle shedding is visible using high-resolution differential interference contrast microscopy, but not in fluorescent microscopic images of stained DNA. It is a challenging task to pinpoint distinct cell morphologies that can be rigorously quantified, provide their mathematical characterization, and use it to develop an algorithm for an automatic image annotation.

As a consequence, the currently available software solutions have a number of drawbacks which limit their usability. Computer programs based on machine learning require laborious manual preparations of large training data sets and are often difficult to use for users without a computer science background. Some of those programs annotate ROIs only using bounding boxes instead of a pixel-wise detection. This allows for a simple counting of NETs and cells, but not for more detailed analyses of their shapes and areas. On the other hand, the currently available tools based on digital image processing, which are free from many of those limitations, quantify NETs, but not the numbers of neutrophils at different stages of NET formation. One of the reasons for this situation is that they are arguably more difficult to develop. While machine learning algorithms, given a manually annotated data set, are able to figure out the crucial steps of image annotation by themselves, tools based on digital image processing techniques need an explicit, human-designed algorithm for this task. Developing such an algorithm requires an in-depth expert knowledge of the analyzed process and dedicated studies on how to mathematically describe and distinguish different cell morphologies.

### 1.3 Trapalyzer: a new computer program to analyze the dynamics and rates of NET formation

**Figure 2:**
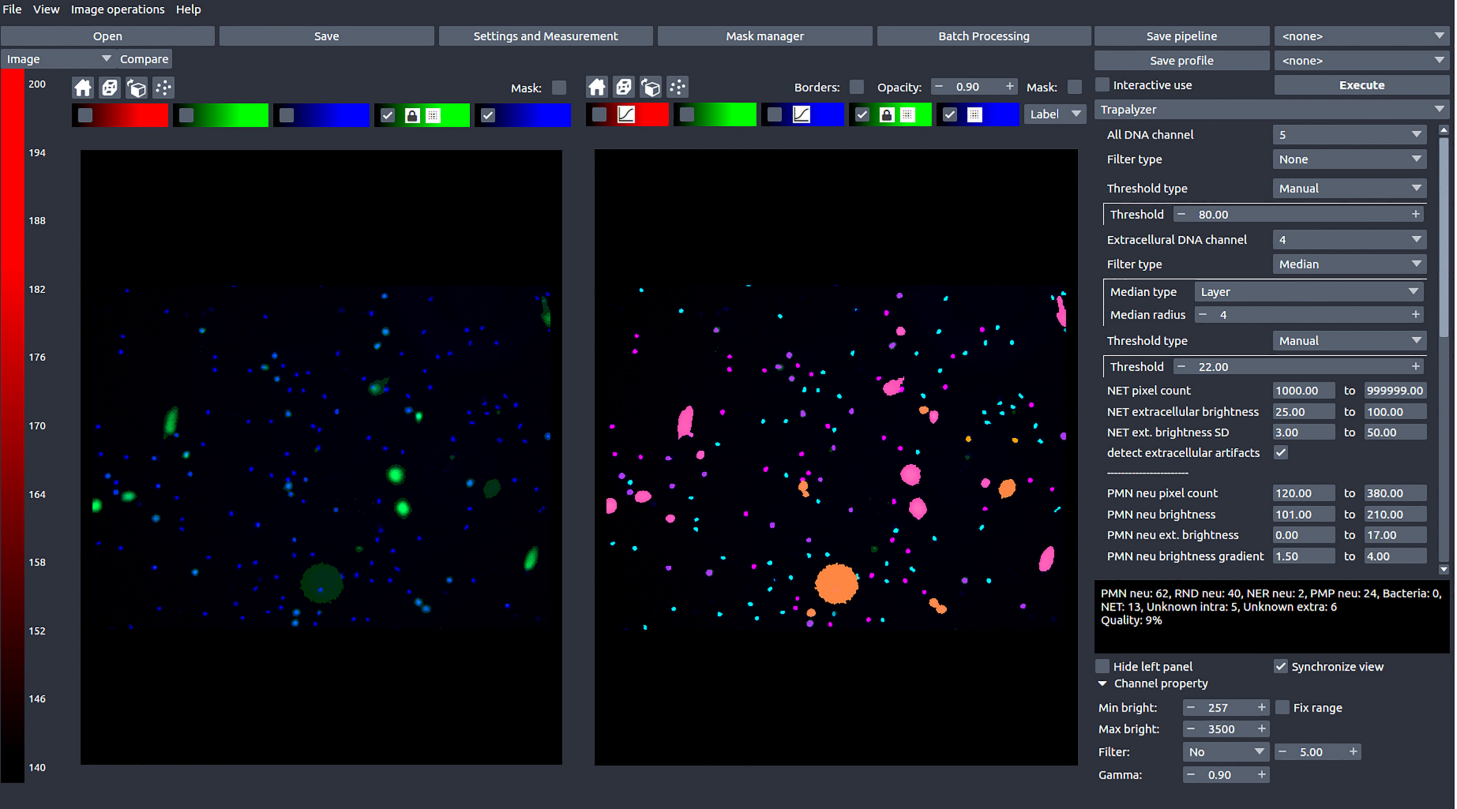
A screen shot of Trapalyzer running in an interactive session of PartSeg. The left window shows a fluorescence microscopy image taken as a part of our neutrophil killing assay with a double staining with SYTOX™ Green and Hoechst 33342 DNA dyes. The right window shows the image annotated by Trapalyzer.

In this work, we present Trapalyzer (**Figure 2**), a computer program for the analysis and annotation of fluorescent microscopy images of neutrophils and NETs double-stained with a cell-permeable and a cell-impermeable fluorescent DNA dye, such as a combination of Hoechst 33342 and SYTOX™ Green. Our software extends the capabilities of the currently available tools by quantifying more stages of NET formation without the need for large training data sets. This has been made possible by extensive studies of fluorescent microscopy images by an interdisciplinary team composed of clinical scientists, statisticians, and computer scientists, which have resulted in a small set of ROI features that characterize the stages, and a scoring system that uses those features to classify cells. To make NET quantification more reliable and robust, the program also detects artifacts in the green channel which can be caused e.g. by background signal or autofluorescence (18). Trapalyzer is freely available as a plug-in for the PartSeg software (19). It can be easily combined with other PartSeg’s features, such as image pre-processing and feature extraction, which further increase the software’s usability.

### 1.4 High-throughput computational analysis of thousands of cells with a user-friendly software

Trapalyzer offers two modes of analysis: an interactive session and a batch processing mode. The interactive session allows the user to set the program’s parameters and visualize the annotation, while the batch processing mode can be used to process multiple images in a single run and save the results in a convenient Excel spreadsheet. The user can specify the information to be computed, both image-wise (such as the number of neutrophils at a given stage of NET formation, the percent of image area covered by NETs, or the quality of annotation) and ROI-wise (such as the area of each ROI, its bounding box, or assigned class). Since Trapalyzer detects ROIs on a pixel-wise basis instead of simple bounding boxes only, it can also calculate multiple different features describing their morphologies.

Trapalyzer is designed with an emphasis on software ergonomy and ease of use. The plug-in requires no installation other than downloading and placing in the PartSeg’s directory and is accompanied with easy to follow tutorials available on the project’s website. The tutorials guide the users through a step-by-step procedure to tune the program’s parameters and configure its output. This allows the users to easily learn how to use the software and apply it to their own experiments even if they have no background in computer science, giving Trapalyzer the potential to be routinely used in laboratories researching diverse aspects of NET formation.

### 1.5 Quantitative analyses of NET formation in different experimental conditions

We validate our approach on a publicly available benchmark data set of neutrophils stimulated with peroxynitrite published in (12) and show that it attains a similar performance to convolutional neural networks using just a fraction of the training data set. We then show how Trapalyzer can be applied to an experiment on the dynamics of neutrophil-*E. coli* bacteria interactions, where we study the cells’ progression through the stages of NET formation. The results of this experiment agree with observations made for individual cells by other authors (8).

## 2 Methods

To establish the quantifiable classes of ROIs for NET formation studies, we have performed a neutrophil killing assay of *Escherichia coli* bacteria. To verify our conclusions and to assess the performance of our approach we have downloaded a benchmark set of images in which neutrophils were incubated without bacteria and NET formation was induced by various chemical stimuli.

### 2.1 Reagents

Roswell Park Memorial Institute (RPMI) 1640 medium, HEPES, SYTOX™ Green, and Hoechst 33342 were purchased from Thermo Fisher Scientific (Waltham, USA). LB broth was purchased from Sigma Aldrich (St Louis, MO, USA).

### 2.2 Preparation of blood neutrophils

Neutrophils were obtained from peripheral blood of one healthy blood donor. Blood sample was purchased at Local Blood Donation Centre and according to local regulations, the blood donor enabled blood donation center to sell their blood samples for scientific purposes and the consent of bioethical committee was not required. Blood was collected into a citrate tube and processed within 2 hours from collection. Neutrophils were isolated using density gradient centrifugation followed by polyvinyl alcohol sedimentation, exactly as described in (20). Isolated neutrophils were suspended in RPMI 1640 medium with 10 mM HEPES (RH).

### 2.3 Preparation of bacteria

*Escherichia coli* (American Type Culture Collection(ATCC) 25922 strain) were grown overnight in LB broth with shaking. In the morning, an aliquot of bacterial culture was taken, diluted 100 x in a fresh LB medium and grown for subsequent 2-3 hours. Subsequently, bacterial cultures were washed and resuspended in RH medium.

### 2.4 Co-culture of neutrophils with bacteria

Neutrophils were seeded into the wells of 48-well plates at the density of 2×10^4^ cells/well and allowed to settle for 30 minutes at 37°C, 5% CO2. Subsequently, *E. coli* was added into the appropriate wells at the multiplicity of infection of 4 or 1 (*E.coli*: neutrophil). Neutrophils incubated without bacteria were used as a control group. A technical duplicate for each condition was prepared. For each intended timepoint (t=0, 60, 90, 120, 180 minutes), a separate 48 well plate was prepared. The plates were centrifuged for 5 minutes at 250 g to allow the contact of bacteria with neutrophils. The plates were incubated at 37°C, 5% CO2 for a specified time and then the samples were stained with SYTOX™ Green (100 nM) and Hoechst 33342 (1.25 µM for 10 minutes. Four images of each well were taken with Leica DMi8 fluorescent microscope equipped with a 10× magnification objective (Leica, Wetzlar, Germany). Overall, 120 images have been obtained.

### 2.5 Benchmark data set

A benchmark data set of images of neutrophils and NETs stained with SYTOX™ Green and Hoechst 33342, published in (12), was downloaded from https://github.com/krzysztoffiok/CNN-based-image-analysis-for-detection-and-quantification-of-neutrophil-extracellular-traps on May 19, 2019. For evaluation of Trapalyzer’s accuracy, we have selected the validation set in file large_validation_set.zip, subdirectory xml_pascal_voc_format/images/oryg. The validation set consists of 57 images. Manual annotations of the images were accessed in subdirectory xml_pascal_voc_format/annotations/oryg. Annotations in xml files were handled using the lxml library of the Python 3 programming language. For tuning of Trapalyzer’s parameters, additional 10 images were selected from the file orig inal_uncompressed_images_with_pascalvoc_annotations.zip, rescaled to match the dimentions of the validation set images and converted to the TIFF format using the convert program from the ImageMagick suite.

## 3 Results

### 3.1 Quantifiable stages of NET formation

In order to pinpoint the stages of NET formation that are suitable for quantification using an automated algorithm, we first analyzed manually a set microscopic images of a neutrophil-*E. coli* co-culture.

#### 3.1.1 Stages of NET formation identified with high-resolution time-lapse microscopy can be observed in low-resolution fluorescent microscopy

**Figure 3:**
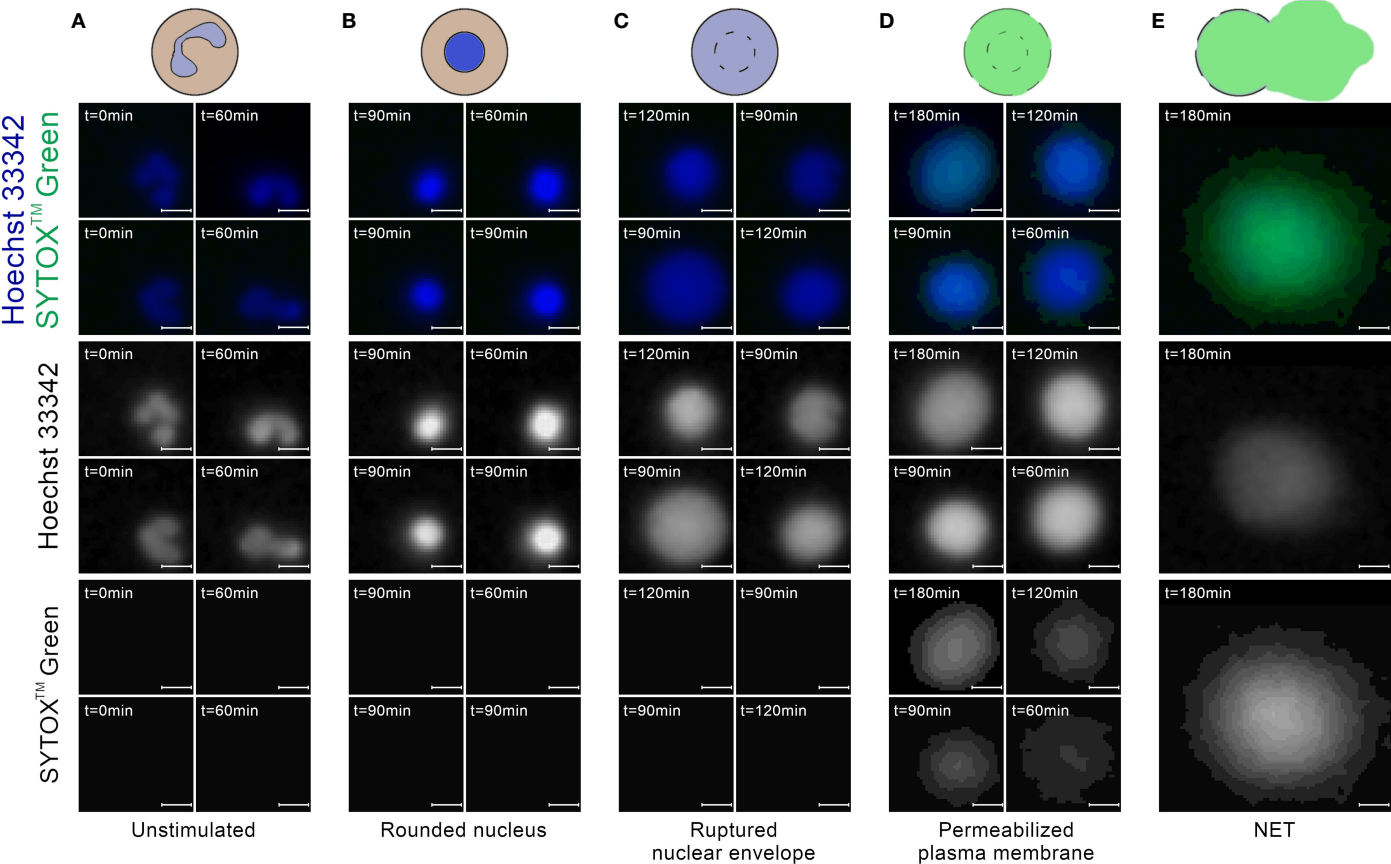
Neutrophils at different stages of NET formation visible in fluorescent microscopy with SYTOX™ Green/Hoechst 33342 double staining. The source images were taken as a part of the neutrophil *E coli* co-culture study. **(A)** Polymorphonuclear (unstimulated) neutrophils; **(B)** Neutrophils with rounded nucleus; **(C)** Neutrophils with ruptured nuclear envelope; **(D)** Neutrophils with permeabilized plasma membrane; **(E)** An example of a neutrophil extracellular trap. Scale bar = 10µm, microscope magnification 100x.

Most of the cells in the images taken at t=0 min exhibited a typical appearance of unstimulated, polymorphonuclear neutrophils, without detectable signal in the extracellular channel (**Figure 3A**). In images taken between t=60 and t=120 min, we have observed cells which were visibly brighter and highly circular (**Figure 3B**). This morphology most likely corresponded to cells with a rounded nucleus. Individual cells exhibited this morphology in t=0 min as well. We have also observed ROIs with larger areas, lower brightness, and cloud-like appearance, with no signal in the extracellular channel (**Figure 3C**). We assume that this morphology corresponded to cells with a ruptured nuclear envelope. We did not observe such cells in t=0 min. In images taken after t=60 min, and mostly in the later stages of the experiment, we have observed cells with cloudy appearance and detectable signal in the extracellular channel (**Figure 3D**). This morphology corresponded to cells with a permeabilized plasma membrane. The intensity of signal in the extracellular channel varied highly for those cells, indicating a gradual permeabilization, in agreement with (8). Throughout the experiment, we have also observed ROIs with significantly larger areas than individual cells, with intense signal in the extracellular channel and detectable signal in the total DNA channel (**Figure 3E**). This morphology corresponded to NETs.

#### 3.1.2 Not all stages of NET formation are suitable for an automatic quantification

Unstimulated neutrophils, cells with rounded nuclei, cells with ruptured nuclear envelopes and cells with permeabilized plasma membranes have distinct morphologies in fluorescent microscopy images, making them suitable for automatic detection by a software tool. On the other hand, neutrophils inbetween those stages, in particular neutrophils undergoing chromatin decondensation and nuclear rounding, are more difficult to classify. The stage of chromatin decondensation (between the onset of NET formation and nuclear rounding) does not seem to have a clear delineation from its surrounding stages with simple qualitative features. As such, this stage does not seem to be a good candidate for a separate class in an automatic classification scheme, at least in fluorescent microscopy images of double-stained DNA. Accordingly, we have decided against distinguishing cells with decondensed chromatin as a separate class of objects. As a consequence, such ROIs were automatically classified as either unstimulated cells or cells with rounded nuclei, depending on the advancement of the NET formation process.

#### 3.1.3 Clumps of bacteria are an important class of ROIs in neutrophil killing assays

**Figure 4:**
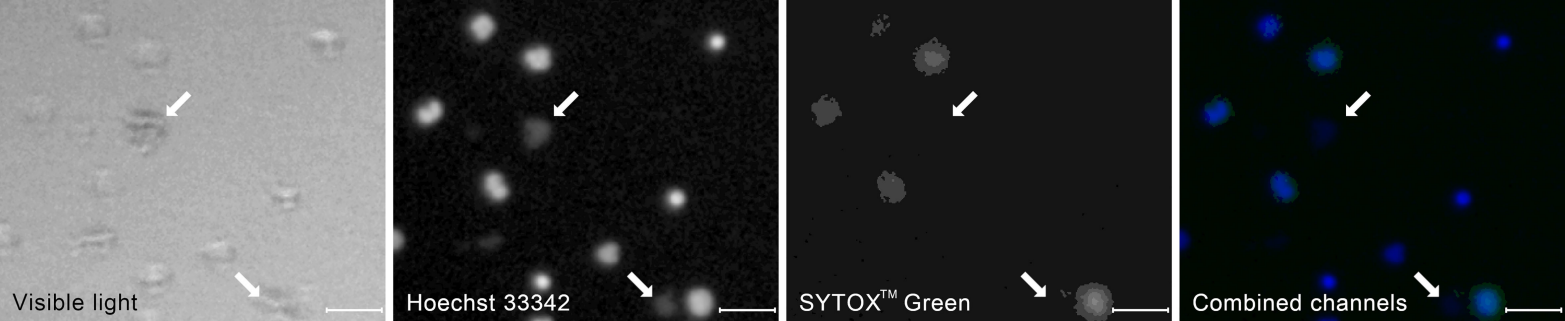
A typical appearance of a clumps of bacterial cells (arrows) in the visible and the fluorescent light compared to the appearance of neutrophil cells at different stages of NET formation. The contrast of the fluorescent images has been enhanced to better visualize the bacteria. The image was taken as a part of the neutrophil-E. coli co-culture study at t=90 min with multiplicity of infection equal 4. Scale bar = 50 µm, magnification 100x. Combined channels: Hoechst 33342 in blue, SYTOX^TM^ Green in green as in **Figure 3**.

Starting from t=120 min, we observed clumps of bacteria, both in the total DNA channel and in the visible light (**Figure 4**). In fluorescent light, they appeared as highly amorphous, low-brightness objects without well-defined edges. With the exponential growth of bacteria, those clumps become prevalent in t = 180 min, motivating the decision to include them as yet another class of ROIs.

#### 3.1.4 Handling artifacts in the extracellular channel can further improve the precision of NET area quantification

**Figure 5:**
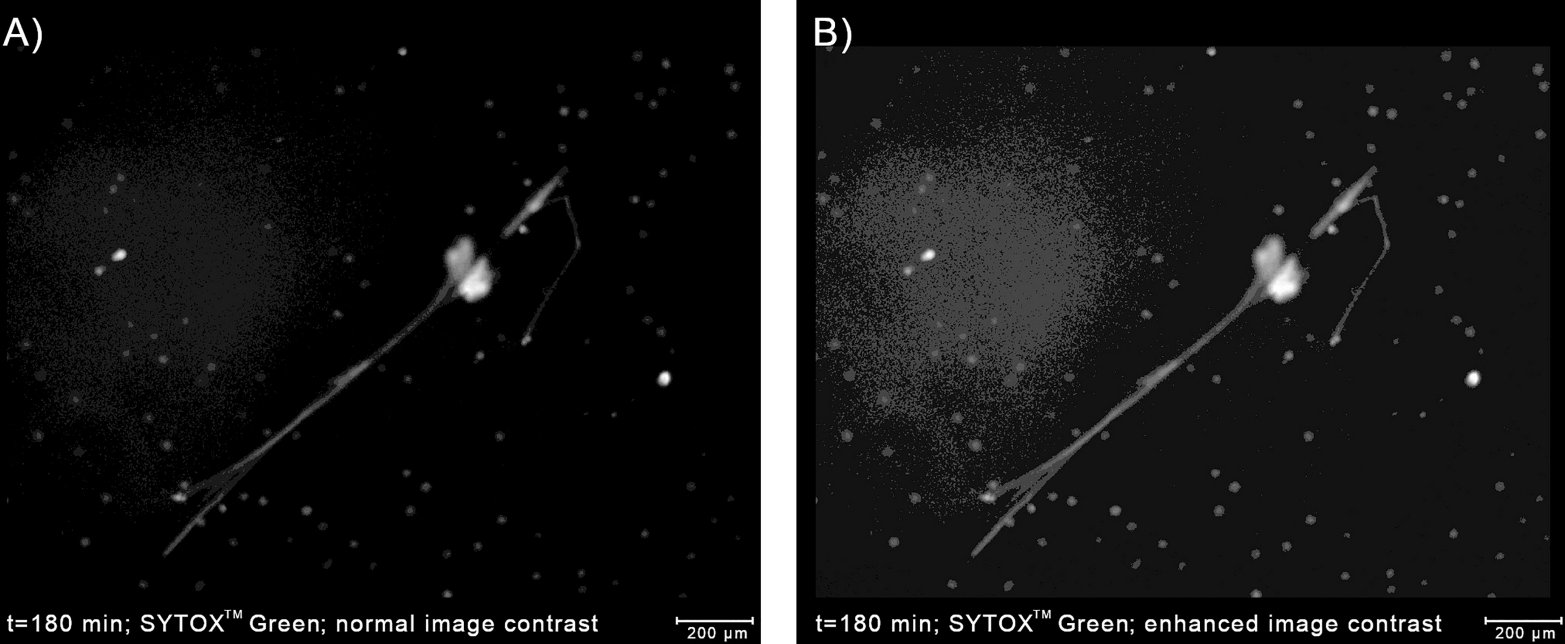
Detecting artifacts in the extracellular channel can improve the accuracy of the determination of the boundaries of NETs. **(A)** An example of a NET with low-brightness regions caused by low chromatin density. Using only the high-brightness regions would underestimate the area occupied by the NET. **(B)** Enhancing the image contrast highlights the NET boundaries, but also reveals a slightly uneven background brightness. Without a proper implementation in the classification algorithm, background regions with a higher brightness can be a source of errors. The image was taken as a part of the neutrophil-*E. coli* co-culture study at t=180 min with multiplicity of infection equal 1.

At the boundaries of NETs, where the chromatin density is low, the fluorescent signal tends to be low as well, making the classification difficult even for human experts (10). Although the low-intensity regions of NETs can be highlighted by increasing the brightness and contrast of the image in the pre-processing stage, the brightness of some regions of NETs can be roughly similar to the background level. On the other hand, the background brightness level itself can be slightly uneven due to e.g. varying plastic density, plastic autofluorescence, or proximity to the edge of the well. As a consequence, increasing the image brightness can result in the appearance of artifacts that can be mistaken for NETs by automatic classifiers (**Figure 5**). When we increased the brightness to capture all the detectable NET regions, we have observed such artifacts in approximately 15% of our images. Therefore, if NETs are to be labeled precisely and reliably in a pixel-wise manner, a NET quantification algorithm should be able to detect and signal potential artifacts in the extracellular channel. On the other hand, if the precise determination of NET boundaries is not needed, the image brightness can simply be adjusted so that artifacts do not jeopardize the analysis. In this case, detection of artifacts may be unnecessary and, accordingly, Trapalyzer allows the user to disable this feature.

Additionally, we have observed individual artifacts in the green channel (with no detectble signal in the Hoechst 33342 channel) with the size of one to a few neutrophil cells. These artifacts may have been caused by out-of-focus cells with permeabilized plasma membrane or a slight contamination with pollen grains. However, since such objects jointly constituted less than 0.1% of ROIs in all images, they did not pose a risk of biasing the results of automatic classification and could be safely ignored.

### 3.2 ROI features at different stages

Based on the results presented in (8) and our analysis of fluorescent microscopy images, we consider seven classes of ROIs: unstimulated neutrophils; cells with decondensed chromatin and rounded nuclei; cells with ruptured nuclear envelopes; cells with permeabilized plasma membranes; neutrophil extracellular traps; clumps of bacteria; and artifacts in the extracellular channel. After fixing the set of ROIs that could potentially be quantified in fluorescent microscopy images of double-stained DNA, we looked for a minimal set of features that could be used to distinguish them.

#### 3.2.1 Stages of NET formation have characteristic values of three ROI features

Polymorphonuclear neutrophils could be distinguished from other classes by a relatively small size and average brightness on the total DNA channel and the lack of signal inthe extracellular DNA channel. Neutrophils with rounded nuclei could be distinguished from other ROIs by particularly high values of average brightness in the total DNA channel. Neutrophils with ruptured nuclear envelopes typically had larger areas than both previous classes. In some cases, they had similar areas to neutrophils with rounded nuclei, but could be distinguished from this class by lower average brightness. Neutrophils with permeabilized plasma membrane covered the same range of areas and brightness in the total DNA channel as the three previous classes. However, they could be easily distinguished from other cells as the only ones with detectable signal in the extracellular DNA channel.

#### 3.2.2 Standard deviation of extracellular signal separates artifacts in the extracellular channel from NETs

Large areas and intense signal in the extracellular DNA channel distinguished NETs from cells, but not from artifacts in the extracellular channel caused e.g. by uneven background brightness. However, due to differences in chromatin density, the brightness of NETs was spatially non-homogeneous, while for the artifacts it was mostly uniform, especially after applying a median filter. This was effectively captured by setting a threshold for the standard deviation of brightness of NETs in the extracellular DNA channel. Notably, with this approach, any ROI that does not conform to the characterization of a NET is classified as an artifact, regardless of its physical origin.

We did not observe an increase in classification accuracy when we additionally included an upper threshold for ROI circularity of NETs, as proposed by other authors (14). On the contrary, NETs can be highly circular in shape (**Figure 3**), especially when formed in the absence of bacteria (12).

#### 3.2.3 Laplacian of Gaussian values characterize clumps of bacteria

The most defining feature of bacterial clumps was the low, highly non-homogeneous signal in the total DNA channel. However, the standard deviation of brightness failed to distinguish them from other classes of ROIs. Another characteristic feature was the lack of well-defined borders, which was effectively captured by small values of the average Laplacian of Gaussian (LoG) of the total DNA channel.

Five features distinguish eight classes of ROIs. The progression of NET formation, the observed morphologies of different stages in fluorescent microscopy images, and the mathematical features that characterize them motivate the classification of ROIs into the following classes:

- *PMN neutrophils*, polymorphonuclear, unstimulated neutrophils, with a moderate cell size and brightness, and a lack of signal on the extracellular DNA channel;
- *RND neutrophils*, cells with decondensed chromatin and rounded nucleus, with higher brightness than PMN neutrophils;
- *RUP neutrophils*, neutrophils with ruptured nuclear envelope, with larger cell sizes than RND neutrophils and possibly lower brightness than PMN neutrophils;
- *PER neutrophils*, neutrophils with a permeabilized plasma membrane, with detectable signal on the extracellular DNA channel;
- *NETs*, Neutrophil Extracellular Traps, with an intense signal on the extracellular DNA channel, low to no signal on the total DNA channel, and noticeable standard deviation of the brightness;
- *Groups of bacteria*, with low brightness and LoG values in the total DNA channel, and possibly moderate signal on the extracellular DNA channel due to possible co-localization with NET fragments;
- *Artifacts in the extracellular channel*, With a moderate, mostly homogeneous signal in the green channel;
- *Unclassified ROIs*, on the intracellular channel, not matching any of the previous classes. This class includes any potential artifacts in the intracellular channel.

Note that the set of features is smaller than the set of classes thanks to a combinatorial approach to class characterization.

### 3.3 Classification workflow

**Figure 6:**
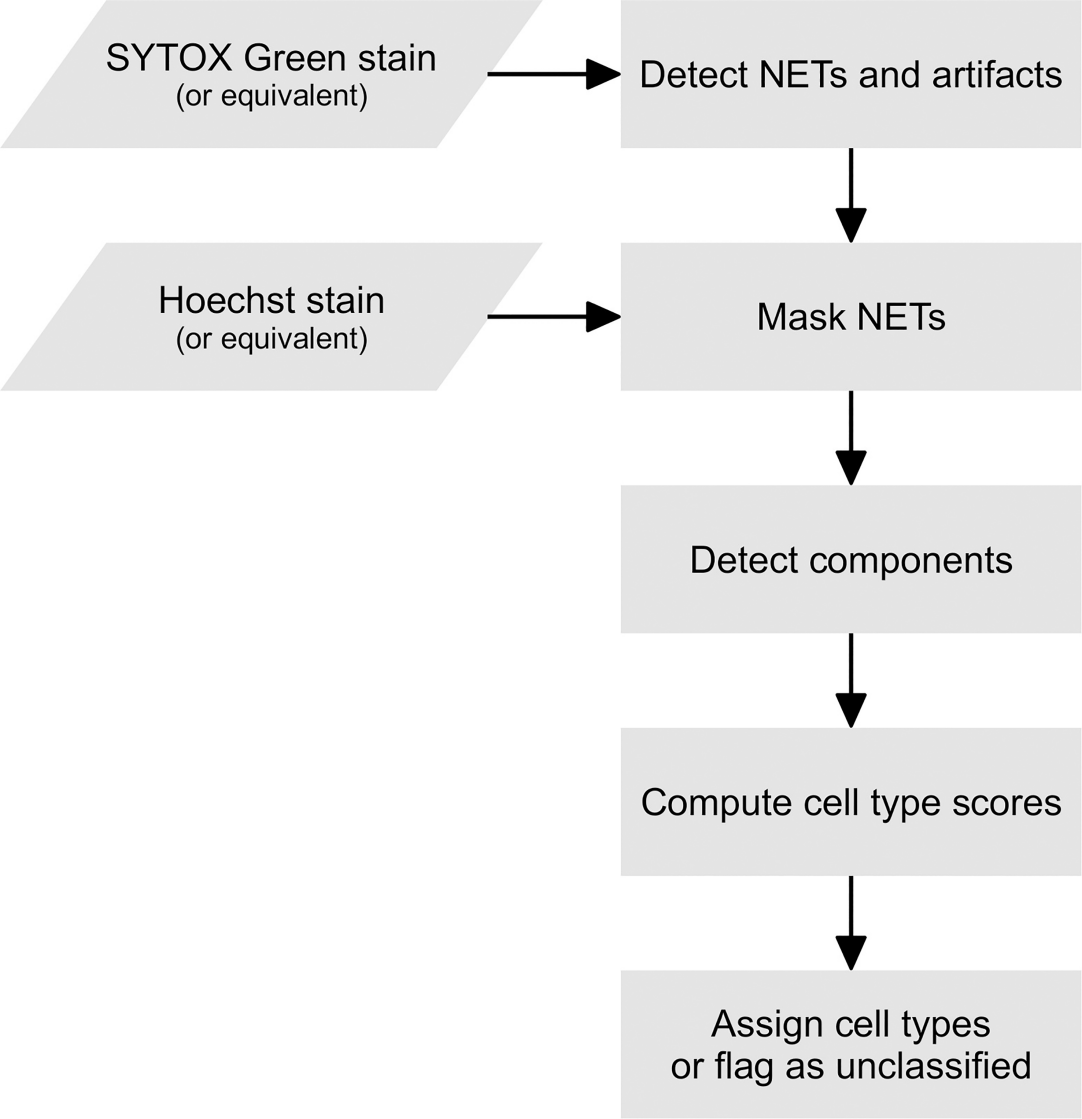
A flowchart of Trapalyzer.

The processing of a single fluorescent image with two channels (an extracellular DNA channel and a total DNA channel) is represented schematically in **Figure 6**. In the pre-processing stage, the user may decide to use one of a number of filters provided by PartSeg (including the Gaussian and the median filter) on any or both channels. In the subsequent ROI detection stage, segmentation is performed on both channels by simple thresholding and detecting connected components. The brightness thresholds for both channels can be adjusted by the user during an interactive session of PartSeg to obtain a segmentation that matches a manual annotation.

In the first classification stage, Trapalyzer classifies ROIs on the extracellular DNA channel. Small ROIs on the extracellular channel, which typically correspond to neutrophils with a permeabilized plasma membrane, are not processed at this stage. If an ROI has a sufficient size and its average brightness and standard deviation are within user-defined ranges, it is classified as a NET. Otherwise, it is flagged as an “extracellular unknown” class, corresponding e.g. to artifacts caused by autofluorescence, uneven brightness, or atypical NETs that require manual inspection.

In the second classification stage, Trapalyzer classifies ROIs in the total DNA channel. For each class, Trapalyzer computes a score that measures whether a given ROI matches its user-defined characterization. The general idea behind the class score is to ensure that all of the ROI features are within appropriate ranges for this class, with an error margin that allows some flexibility when defining the ranges.

Formally, let *x* are the value of a particular feature (e.g. brightness) of a given ROI, and let [*l*,*u*] are the acceptable interval for this feature for a given class of ROIs (e.g. RUP neutrophils). For a single feature, we define a partial score function *S*(*x*;*l*,*u*,*s*) where the *s* parameter controls the extent of the error margins. The idea behind the partial score function is that *S*(*x*;*l*,*u*,*s*) equals 1 if x∈[*l,u*] and falls smoothly to 0 is *x* becomes distant from the interval [*l,u*] with the decrease rate controlled by *s*. Formally, we want *S* to be equal to 0 either when $x\leq l-sl\frac{u-l}{u+l}=l-{\text{Δ}}_{l}\left(s\right)$ or $x\geq u+su\frac{u-l}{u+l}=u+{\text{Δ}}_{u}\left(s\right)$ . This way, the left and right error margins for the characteristic range, ${\text{Δ}}_{l}\left(s\right)$ and ${\text{Δ}}_{u}\left(s\right)$ , adjust to the interval length and its boundary values. Because of this, a single value of the error margin parameter *s* can be set for all features regardless of their units and typical values.

The properties described above are satisfied by the following function, where $l_{0}=l-{\text{Δ}}_{l}$ and $u_{0}=u+{\text{Δ}}_{u}$ define the range in which we want *S* to have a non-zero value:


$$S\left(x;\;l,u,s\right)=\{\begin{array}{ccc} 1 & \text{if} & x\in \left[l,u\right]\\ \frac{1}{2}+\frac{1}{2}\text{sin }(\pi \frac{x-l+\Delta_{l}}{\Delta_{l}}-\frac{\pi }{2}) & \text{if} & x\in \left[l_{0},l\right]\\ \frac{1}{2}+\frac{1}{2}\text{sin }(\pi \frac{x-u+\Delta_{u}}{\Delta_{u}}-\frac{\pi }{2}) & \text{if} & x\in \left[u,u_{0}\right]\\ 0 & \text{otherwise} \end{array}$$

To obtain a final score for a given class, we multiply the partial scores for all the features of the analyzed ROI. This ensures that an ROI fully matches a class when all the features are within or sufficiently close to their acceptable ranges.

An ROI is determined as belonging to a given class if the score for this class is sufficiently high (by default above 0.8), and the scores for all the other classes are sufficiently low (by default below 0.4). The upper threshold for the scores of “competing” classes ensures that an ROI is classified unambiguously. If an ROI does not reach a sufficiently high score for any class, or reaches a high score for more than one class, it is flagged as an unknown class that requires a manual inspection.

During the second classification stage, we mask the regions occupied by NETs and artifacts in the extracellular channel and ignore ROIs in those regions. This is because, if a neutrophil lies within such a region, it is either difficult or impossible to accurately distinguish whether or not it has a permeabilized plasma membrane, and classification would therefore be unreliable. On the other hand, the number of such ROIs is typically small compared to the overall number of ROIs, and ignoring them had a relatively small influence on the overall performance of the software.

After both classification stages are completed, Trapalyzer evaluates the quality of image annotation. We define the quality score as Q=100·(1−U/S)% , where *U* denotes the area covered by unclassified ROIs and *S* denotes the area covered by all detected ROIs. We use the areas of ROIs instead of their numbers to make the score robust to small artifacts that otherwise do not interfere with the analysis.

### 3.4 Validation on a benchmark data set

In order to assess Trapalyzer’s accuracy, we have compared it to previously reported results achieved with convolutional neural networks (CNNs) on a publicly available benchmark data set (12). To match the original study, we have restricted the classification to four classes: PMN, RUP and PER neutrophils and NETs (the RUP neutrophils were referred to as *decondensed* in the original work).

First, we tuned the parameters on 10 images selected from a training data set. Then, we used Trapalyzer’s batch processing mode to analyze a full validation data set of 57 images containing 1083 manually annotated objects. We compared the resulting annotation with the manual one provided with the data set. We used an Intersection over Union (IoU) threshold of 0.10, meaning that we match objects if the overlap of their bounding boxes is at least 10% of their joint area. The IoU value was based on previous results in NET quantification (10, 12). In case of more than two ROIs with overlapping bounding boxes, the pair with the highest IoU was selected as a match.

#### 3.4.1 Consistent image acquisition conditions are crucial for automated image analysis

**Figure 7:**
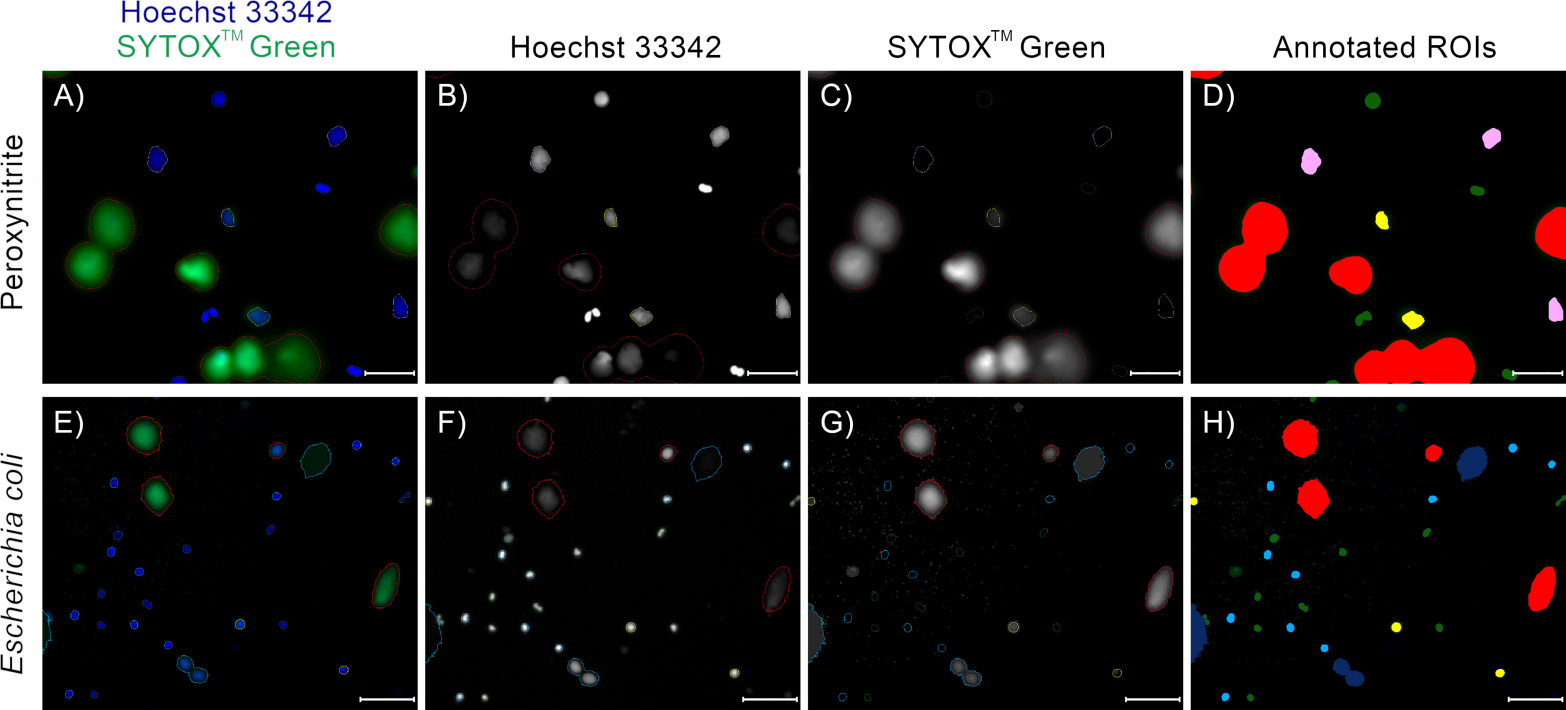
Examples of annotations of fluorescent microscopy images of NETs and neutrophils double-stained with Hoechst 33342 and SYTOX™ Green, with neutrophils acquired from different patients, NET formation induced with different stimuli, and different microscope magnifications. **(D, H)** Trapalyzer identified the following classes of objects: Red: Neutrophil Extracellular Traps; Green: PMN (polymorphonuclear) neutrophils; Light blue: RND (rounded nuclei) neutrophils; Pink: RUP (ruptured nuclear envelope) neutrophils; Yellow: PER (permeabilized plasma membrane) neutrophils; Dark blue: artifacts in the extracellular channel. **(A–D)** A fragment of an image from the benchmark data set (12). NET formation was triggered with 100 µM peroxynitrite. Scale bar = 75µm, magnification 400x. **(E–H)** A fragment of an image taken as a part of the neutrophil-*E. coli* co-culture study at t=120 min with multiplicity of infection equal 1. Scale bar = 125µm magnification 100x. In this example, two closely located emerging NETs in the bottom part of the image were flagged as a potential artifact for manual inspection due to low standard deviation of brightness in the extracellular channel. Lowering the standard deviation threshold results in their proper classification as NETs.

The results of Trapalyzer annotation are shown in **Table 1**, and an example annotation is shown in **Figure 7A**. On average, 11.90% of ROIs were unclassified in each image, with two images exceeding 50% due to atypically low brightness of cells, likely caused by a low exposure time.

**Table 1:** The results of an analysis of a publicly available benchmark data set of 57 fluorescent microscopic images.

		Ground truth (manual annotation)						
		NET	PMN neu	RUP neu	PER neu	Unmatched	Total predicted	Precision
Trapalyzer	NET	223	1	0	5	2	231	0.97
	PMN neu	0	365	3	0	14	382	0.96
	RUP neu	0	0	34	0	0	34	1.00
	PER neu	16	0	4	200	5	225	0.89
	Unknown	1	31	37	4	59	132	N/A
	Unmatched	66	2	0	11	0	79	N/A
	True total	306	399	78	220	80		Avg=0.95
	Recall	0.73	0.91	0.44	0.91	N/A	Avg=0.75	

N/A, Not Applicable.Shaded values: NETs and neutrophil cells. Values in bold: correctly classified ROIs.

#### 3.4.2 Simple classification workflow achieves precision on par with convolutional neural networks

Over all ROI classes, Trapalyzer achieved an average precision of 95%, higher than 91% reported for the CNN classifier trained on 188 images. Precision varied slightly between classes, with the highest equal 100% for RUP neutrophils, and the lowest equal 89% for PER neutrophils, caused by annotating some NETs as neutrophils with permeabilized plasma membrane. Separating NETs from PER neutrophils is difficult due to the gradual nature of chromatin release into the extracellular environment, so mismatches between the manual and automatic annotation are to be expected.

#### 3.4.3 Trapalyzer avoids uncertain classifications

We have achieved an average recall of 75%, lower than 93% reported for the CNN. However, the recall varied greatly between classes, from 44% for RUP neutrophils up to 91% for PMN and PER neutrophils. Approximately half of the RUP neutrophils were flagged as an unknown ROI class, suggesting imperfect parameter estimation from the training data set. This stands in agreement with a philosophy that when parameters are misspecified, it is safer to avoid classifying ROIs than to classify them wrong. Note that, in practice, it is always possible to fine-tune the parameters on additional images to increase the recall.

#### 3.4.4 Quantifying NET area is more reliable than NET count

The recall value for neutrophil extracellular traps was 73%, caused by difficulties with matching Trapalyzer and manual annotations. Inspecting selected images showed that Trapalyzer missed fragments of NETs with a low brightness (on the verge of the background signal), causing low IoU values due to large differences between detected and manually generated bounding boxes. Moreover, NETs tend to merge if released by closely located cells and, although a human expert can detect such cases and identify individual nets, our approach to segmentation treats them as a single object. This agrees with observations made by other authors that the numbers and areas of individual NETs are difficult to quantify algorithmically, and quantifying the total image area covered by NETs is more reliable (10).

### 3.5 A detailed analysis of a neutrophil-E. coli co-culture

As an example application of Trapalyzer, we have performed a detailed analysis of the 120 fluorescent microscopy images of neutrophils incubated with or without *E. coli* bacteria, which we used to establish quantifiable classes of ROIs in the previous subsections. We have tuned the software’s parameters on a set of selected 10 images and further adjusted them on images with large numbers of unclassified ROIs. The correctness of annotation was then validated by an expert on 5 images. An example annotation of a fragment of an image is shown in **Figure 7**. In total, Trapalyzer detected and annotated 16924 ROIs, including 10905 polymorphonuclear neutrophils, 733 neutrophils with a rounded nucleus, 266 neutrophils with a ruptured nuclear envelope, 2742 neutrophils with a permeabilized plasma membrane, 344 NETs, 698 clumps of bacteria, 265 artifacts on the SYTOX™ Green extracellular channel, and 971 unclassified components in the Hoechst 33342 total DNA channel.

#### 3.5.1 Population-level results support the current model of NET formation

The total number of cells detected by Trapalyzer stayed approximately constant over the duration of the experiment (**Supplementary Figure S2**), in agreement with the fact that only a few cells release NETs, showing that the experimental conditions and image acquisition methods were consistent. We observed a gradual decrease of the number of PMN neutrophils over time and the transition to RND, RUP, and PER cell morphology types (**Supplementary Figure S1**), in agreement with the observations made for individual cells in (8). NETs were formed continuously throughout the experiment and their number seemed to grow linearly in all experimental conditions, including the control group without bacteria (**Supplementary Figure S2**). However, the rate of NET formation is higher in the co-cultures than in the control, indicating that the presence of bacteria successfully induced NET formation.

#### 3.5.2 ROI-level results suggest an additional stage of NET formation

**Figure 8:**
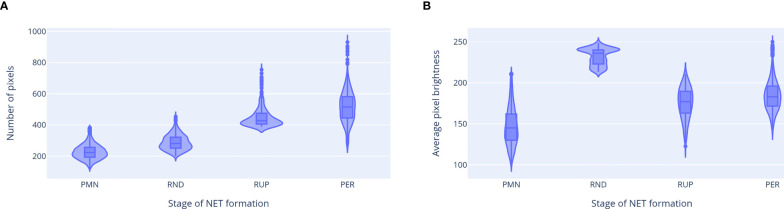
Properties of ROIs classified as neutrophils at different stages of NET formation. **(A)** ROI area (in number of pixels). **(B)** Average pixel brightness in the total DNA channel. The third feature, average brightness on the extracellular channel, had a non-zero value only for the neutrophils with permeabilized plasma membrane. Abbreviations of NET formation stages: PMN, polymorphonuclear; RND, rounded nuclei; RUP, ruptured nuclear envelope; PER, permeabilized plasma membrane.

The properties of ROIs classified as neutrophils at different stages of NET formation are shown in **Figure 8**. Different classes were clearly separated by the ROI features used in our classification workflow, which confirms their distinct natures. We observed a gradual increase in cell size as NET formation progressed. The average brightness was the highest for RND neutrophils, likely due to chromatin decondensation and increased dye affinity, and decreased after the rupture of the nuclear envelopewhen the chromatin occupied a larger area. A visibly bi-modal distribution of the brightness of RND neutrophils suggests that there may be an additional stage NET formation, which causes this group to be composed of two different types of cell morphologies. This phenomenon requires further studies.

#### 3.5.3 Mathematical modeling of the dynamics of a neutrophil population poses additional challenges

Neutrophils stayed viable for a prolonged period of time in the negative control without bacteria. From the perspective of mathematical modeling, this means that traditional models based on ordinary differential equations may not be suitable to describe an *in vitro* cultured population of neutrophils. More sophisticated mathematical techniques,such as delay differential equations, may be required to model the dynamics of NET formation in such experiments.

#### 3.5.4 A small number of false positives did not influence the overall conclusions

The number of bacterial groups grew exponentially in time when bacteria were present in the sample (**Supplementary Figure S2**). This suggests that, in our experimental conditions, neutrophils had a limited capability to eliminate pathogens. We have observed a small number of false positive results in the control group without bacteria. In 8 out of 40 images for this experimental condition there were between one and three improperly detected clumps of bacteria, corresponding to image artifacts and misclassified neutrophil cells. In comparison, in the experimental condition with four bacterial cells per neutrophil, there were up to 100 bacterial groups for t=180 min. As a consequence, the number of false positive detections of bacterial clumps was comparatively small and did not influence the overall conclusions.

## 4 Discussion

Software tools designed for the analysis of microscopic images of neutrophils and neutrophil extrcellular traps (NETs) can be roughly partitioned into two groups. The first group consists of tools based on machine learning, such as convolutional neural networks (CNNs) or support vector machines (SVMs). The second group consists of tools based on classical image processing techniques, such as edge detection, image filtering etc.

Trapalyzer employs the latter approach. It detects ROIs using a thresholding operation and classifies them as NETs, neutrophils at different stages of NET formation, or clumps of bacteria based on a handful of features such as ROI area and average brightness. An ROI is assigned to a class if it matches it unambiguously according to a scoring function.

Our reasons to make Trapalyzer machine learning-free are twofold. First, machine learning algorithms require extensive training data sets on which they learn how to distinguish between different types of objects. Preparing such a data set can take many days for a trained expert. The algorithm is then capable of classifying objects in new images only as long as their morphology closely resembles the objects encountered in the training data set. This is particularly limiting in case of NETs, because their morphologies may differ depending on the experimental conditions, such as the substance used to stimulate neutrophils to release the traps (7). Even minor changes in experimental conditions, such as microscope magnification or exposure time, may require a retraining of the algorithm by a machine learning expert. This negates one of the purposes of such tools, which is to make analyses easier, faster, and less laborsome.

Despite the varying morphology, NETs retain certain characteristic features - large size, signal in the extracellular channel - that can be used to detect and quantify them. Identifying such features and using them in Trapalyzer makes the software more robust to small variations in morphology and more general in terms of applications to different experiments. A change in microscope magnification or exposure time requires only a simple change of a few parameters instead of retraining of the whole algorithm. On the other hand, since the parameter values depend on the equipment used in a particular laboratory, we do not include a default set of parameters in Trapalyzer. Instead, we provide an easy to follow tutorial with a step-by-step procedure of tuning them, available on the project’s website.

The second reason to avoid the use of machine learning algorithms is that they typically operate on a black-box basis, meaning that the way they arrive at their classification is unknown and often too complex to be understood by humans (21). As a consequence, if a neural network misclassifies a given type of ROIs in a given experiment, it is either difficult or impossible to identify why this happens and how to fix it. On the other hand, digital image processing techniques and simplified algorithms used in Trapalyzer provide control over the classification process without sacrificing the precision of the results. The user may freely decide which cell types are of interest in a given experiment and which features to use for classification. If an ROIs is misclassified by Trapalyzer, it is easy to check which parameter of the software has an improper value and adjust it.

In this work, we detect and classify polymorphonuclear neutrophils solely based on their size and average brightness. Due to their characteristic shapes, some measure of ROI circularity could potentially also be a characteristic feature of this morphology. Two common measures of this feature are the ratio of the ROI area to squared perimeter and the ratio of the ROI area to squared diameter. However, none of those measures was capable of distinguishing polymorphonuclear neutrophils from other stages of NET formation and improve the accuracy of classification. This is because some segments corresponding to those cells are elongated but otherwise highly regular, and both circularity measures are high in such cases. We did not find any mathematical characterization of the irregular shape of polymorphonuclear neutrophils that would be useful for our purposes.

Trapalyzer is designed for studies based on double staining of neutrophils with DNA-binding dyes and live imaging of unfixed samples. Despite the characteristic morphology of NETs in such images, their identification based solely on DNA staining may not always be sufficient. This technique is complementary to, but not a substitute of, immunohistological staining which confirms the presence of characteristic proteins ornamenting DNA threads, such as neutrophil elastase, histones, or myeloperoxidase. Immunofluorescent labeling is especially important when novel or uncommon inducers of NET release are studied (12, 13). Nevertheless, once the formation of NETs is confirmed by immunostaining, double-staining of the DNA provides a robust and easy way to quantify the phenomenon of NET release.

To our knowledge, Trapalyzer is the only currently available computer program capable of quantifying not only NETs in terms of their number and area, but also the numbers of neutrophils at different stages of NET formation, in experiments where extensive training data sets are not available. Currently, Trapalyzer can only be applied to cultures of isolated neutrophils. Extending the software capabilities to handle co-incubation of neutrophils with other types of cells, e.g. cancer cells, is a potential direction of future developments.

## Data availability statement

The raw data supporting the conclusions of this article will be made available by the authors, without undue reservation.

## Ethics statement

Ethical review and approval was not required for the study on human participants in accordance with the local legislation and institutional requirements. Written informed consent for participation was not required for this study in accordance with the national legislation and the institutional requirements.

## Author contributions

MC and AG conceived the study. MC, GB and AM-H designed the software. GB implemented the software. MC and AM-H designed the co-culture experiment. AM-H and WK performed the experiment. MC analyzed the experimental data and wrote the draft manuscript. AG and UD supervised the work. All authors have read and approved the manuscript.
