# Supplementary material for: Trapalyzer: a computer program for quantitative analyses in fluorescent live-imaging studies of neutrophil extracellular trap formation
Source: Front Immunol. 2023 Jun 8;14:1021638. doi: 10.3389/fimmu.2023.1021638 (PMC10285529; doi:10.3389/fimmu.2023.1021638)
Supplement: Supplementary file 1 [file DataSheet_1.pdf]

Supplementary Material for Trapalyzer: A computer program for quantitative analyses in fluorescent live-imaging studies of Neutrophil Extracellular Trap formation.

1 SUPPLEMENTARY TABLES AND FIGURES

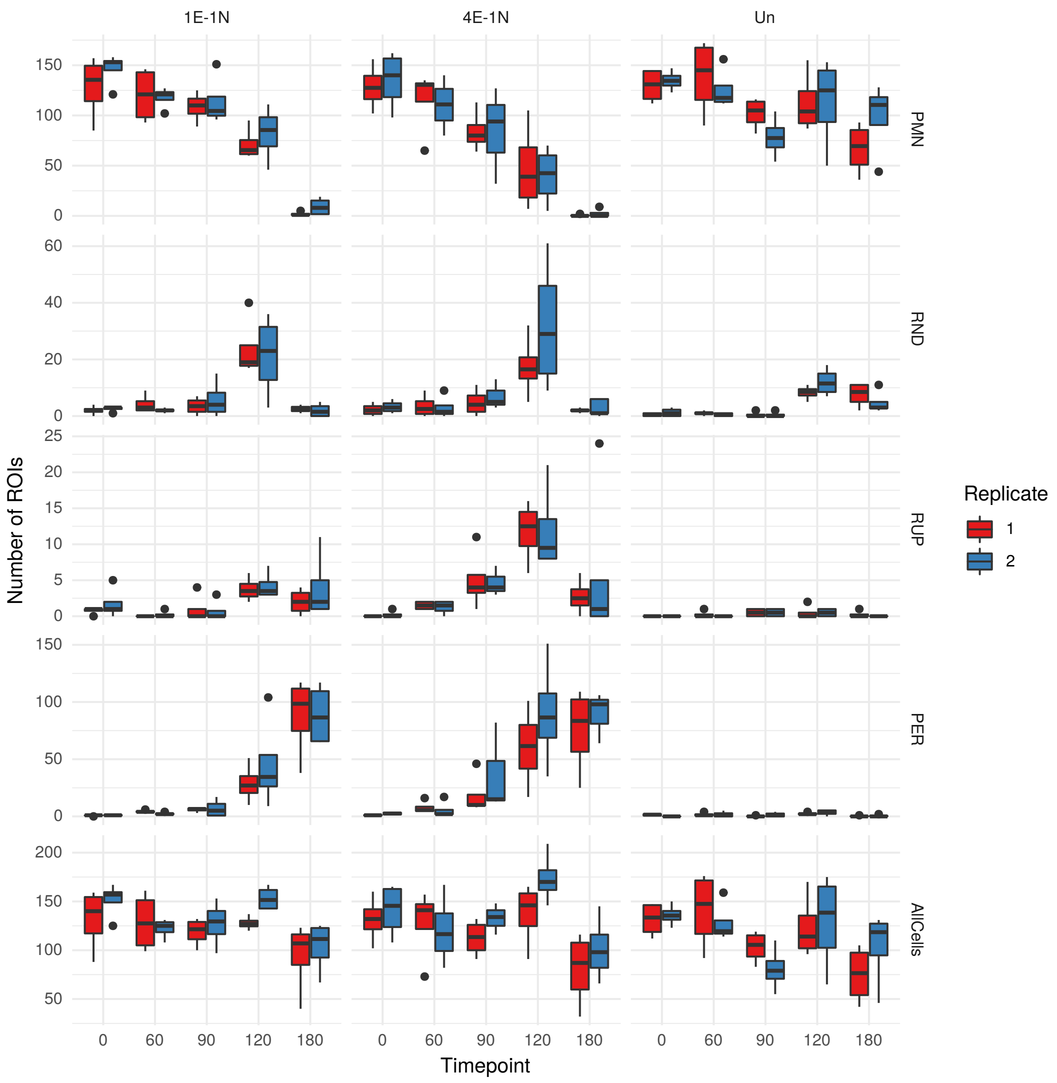

Figure S1. The progression of NET formation in a neutrophil-*E. coli* bacteria co-culture experiment. Each row shows the percentage of cells in a given stage of NET formation. Data was obtained by analyzing 120 fluorescent microscopy images with Trapalyzer. Four images were taken for each timepoint, experimental condition, and technical replicate. Abbreviations of NET formation stages: PMN, polymorphonuclear; RND, rounded nuclei; RUP, ruptured nuclear envelope; PER, permeabilized plasma membrane.

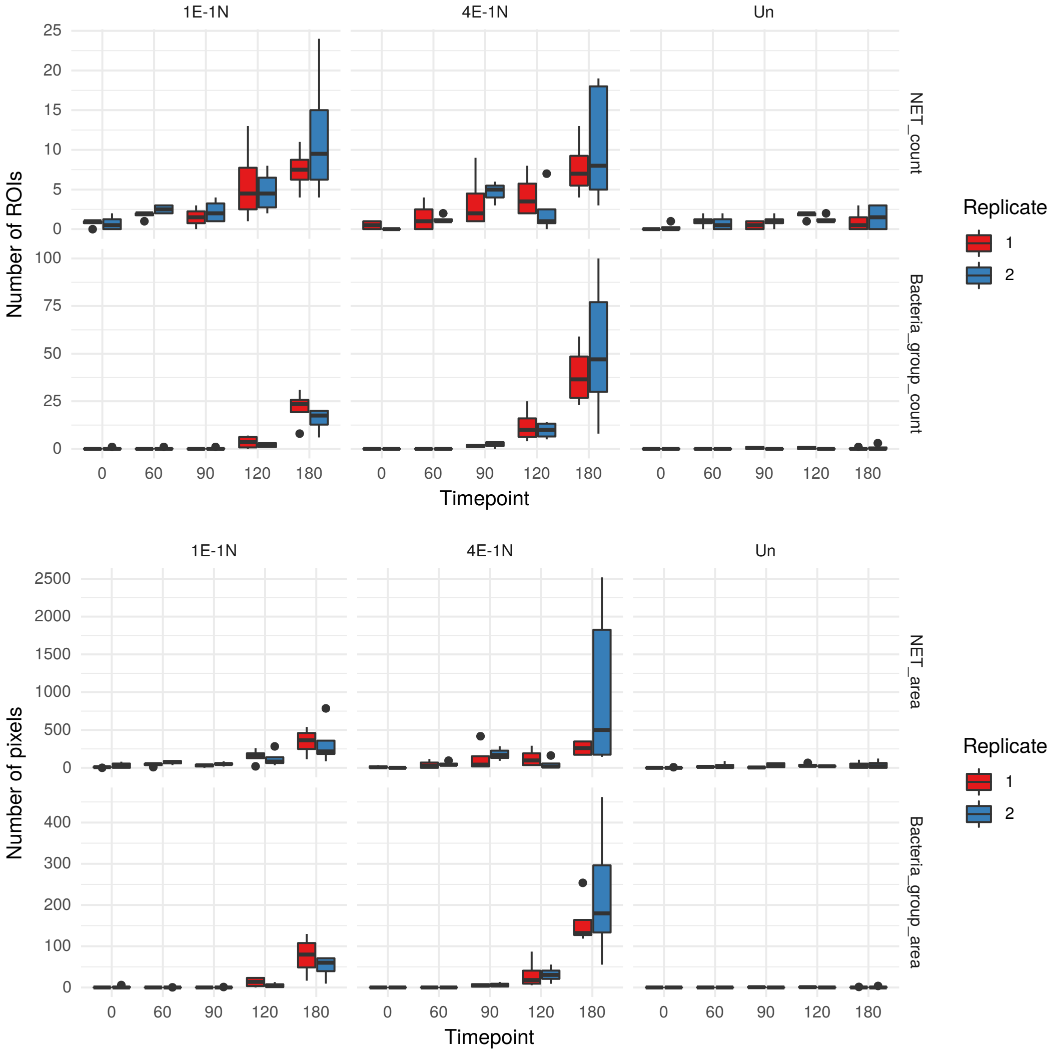

Figure S2. The numbers (top) and areas (bottom) of neutrophil extracellular traps and groups of bacteria, obtained by analyzing fluorescent microscopy images from the neutrophil-*E. coli* co-culture experiment with Trapalyzer.
